# The Living-Related Kidney Transplant Program in Brunei Darussalam: Lessons Learnt from a Nascent National Program in a Small, Muslim, and Asian Country

**DOI:** 10.1155/2021/8828145

**Published:** 2021-04-20

**Authors:** Jackson Tan, Muhammad Abdul Mabood Khalil, Dalinatul Ahmed, Jayakrishnan Pisharam, Chiao Yuen Lim, Hock Beng Chua, William Chong, Kim Khee Tan

**Affiliations:** ^1^Department of Nephrology, RIPAS Hospital, Bandar Seri Begawan BA1710, Brunei Darussalam; ^2^Universiti Brunei Darussalam, Bandar Seri Begawan BA1710, Brunei Darussalam; ^3^Department of Surgery, RIPAS Hospital, Bandar Seri Begawan BA1710, Brunei Darussalam

## Abstract

Brunei Darussalam commenced its living-related renal transplant program in 2013, with subsequent attainment of independent local capacity and proficiency in 2019. The preliminary outcome from the program has already begun to shape the national nephrology landscape with a 36% increment in transplant rate and mitigation of commercialized transplantations. The blueprint for the program was first laid out in 2010 and thereupon executed in four phases. The first phase involved the gathering of evidence to support the establishment of the national program, through researches investigating feasibility, public opinion, quality of life, graft survival, and cost-effectiveness. The second phase focused on laying the foundation of the program through grooming of local expertise, implementation of legal-ethical frameworks, religious legitimization, and propagation of awareness. The third phase worked on facilitating experiential exposure and strengthening local infrastructure through the upgrading of facilities and the introduction of subsidiary services. The fourth phase was implemented in Brunei in 2013 when foreign personnel worked together with the local team to perform the transplants. Between 2013 and 2019, ten kidney transplants were performed, with two being done in 2018 and three in 2019. We hope to inspire other similar countries to develop their own self-sustainable and independent local program.

## 1. Introduction

Despite having the smallest population in Asia [1], Brunei has one of the highest rates of end-stage kidney disease (ESKD) in the world [2, 3]. There were 783 patients on renal replacement therapy (RRT) at the end of 2018 [4]. The latest prevalence and incidence of ESKD in the country were 1769 per million population (pmp) and 380 pmp [4], based on a national population of 442,400 [5]. The corresponding prevalence and incidence of transplant in the country were 104 pmp and 9 pmp [4]. Transplant incidence in the country was very low compared to those in developed countries [3] but on par with rates achieved in neighboring Asian countries such as Singapore (18 pmp), Taiwan (13 pmp), Hong Kong (11 pmp), Thailand (10 pmp), Philippines (5 pmp), and Malaysia (3 pmp) [2]. Like many of these Asian countries, commercialized transplantations contributed significantly to the overall transplant incidence [6]. Prior to the inception of the local transplant program, patients were sent overseas under full government sponsorship for transplants in the United Kingdom, Singapore, and Malaysia, with the first such case in 1978 [7]. A small number of patients also had kidney transplants by illegal means through commercialization [8]. The national living-related transplant program was established in 2013 with the assistance of foreign expertise. By 2019, the local team was able to perform transplants independently.

Literature on establishing a national transplant program was scarce, especially in the settings of a small, Muslim, and Asian country. Due to the country's small population, there have always been reservations about a program's viability and sustainability due to insufficient demands and expertise retention. Funding and support from experienced international associations were less forthcoming because of the affluent economic status of the country and the presumptive conviction that existing pecuniary resolve could prevail over logistical and expertise restrictions. Small countries also have to grapple with the lack of a recognized training program and the “brain drain” phenomenon with suitable foreign-trained doctors seeking work opportunities overseas [9]. Other small southeast Asian countries like Laos, Cambodia, and Timor Leste [10] have piggybacked on transplant services in neighboring countries due to constraints in setting up their own viable programs. Most Asian countries also have unique barriers that hampered kidney donations, particularly related to uncertain cultural beliefs toward transplant, concerns of posthumous disfigurement, and nonexistent regulation about commercialization [11]. In addition, Muslim countries often had to wrangle with issues like religious permissibility of transplant and the concept of brain death [12]. Over and above that, political will and support in Brunei to initiate this process had been tepid and perfunctory in the past few decades. Moreover, insufficient resources had been invested in research to investigate the cost-effectiveness, efficacy, and merits of a local program. Recent transplant successes were reported in larger Asian countries like Myanmar (1997) [13], Armenia (2002) [14], Nepal (2008) [15], Mongolia (2006) [16], Tajikstan (2009) [17], Uzebekistan (2010) [2018], and Kyrgyzstan (2012) [17]. We drew a lot of optimism and encouragement from these programs, and their outcomes are summarized in Table 1.

Not many clinicians have the privilege and experience to start a transplant program in a country, and the task is not helped by the paucity of data available in the literature. We would, therefore, like to share our experience of setting up a national program in a previously transplant naïve country, especially focusing on overcoming issues like the lack of expertise and facilities and religiocultural and political-legislative challenges. We will also describe a case series of our patients and present the impact of the program on our services.

## 2. Materials and Methods

This case series described in detail the ten transplant cases that were performed in the country. The objectives of the study were to share our experience with phases of the establishment of a kidney transplant program and share our results of the first cases done. The duration of the study was from 2013 to the end of 2019 during which 10 kidney transplants were done. Personal patients' data were obtained through personal accounts from clinicians and recorded data in hospital computerized information system. Data collected included patient demographics and data pertaining to the operations, hospital stays, and outpatient follow-ups. Patient demographics included gender, age, the relationship between patients, year of operation, etiology, medication, blood group, crossmatch results, blood results, and radiographic findings. The operation, inpatient, and follow-up data were also collected. These results are presented in Tables 1–3.

Data for the country were obtained from the Brunei Dialysis and Transplant Registry [4] and unpublished registry data from the Department of Renal Services, Ministry of Health (MOH). The collected data included incidence, prevalence, the total number of patients in each of the modalities, and percentage increments per year. In addition, data were also obtained from previous publications from the department [9] and historical records from the MOH. The data that were collected included the year of the transplant, the country where transplants were performed, and sponsorship details of the transplant. Statistical analysis was done with Excel and Shapiro-Wilk test (*p* > 0.05), and a visual inspection of the histogram was done for age, operation time, and warm ischemia time. These data are presented in Tables 4 and 5.

## 3. Results

Table 2 showed the demographic, operative, and hospital stay details of all our ten transplant recipients. The age of the patient was normally distributed. The mean age of recipients was 30.5 ± 9.3 years. The majority of recipients have an unknown cause of ESKD, and only three showed biopsy evidence of chronic glomerulonephritis. Three out of ten recipients had donor-specific antibodies of which two received desensitization prior to the transplant. Operation time was normally distributed, and the mean operation time was 267.7 ± 43.3 minutes. The mean and median warm ischemic time was 112.9 ± 30.4. One surgical reexploration for infected wound hematoma was required. Hospital stay was not normally distributed, and the median hospital stay was 15 days.

Table 3 showed the follow-up details of the recipients. The mean and median discharge creatinine was 106.5 ± 27.5 and 92 mmol/l. All patients had excellent graft function at one year, with all five patients who had two years of follow-up maintaining stable results.  Table 4 showed the demographic, operative, and hospital stay details of all our ten transplant donors. The mean age and median age of donors were 38.6 ± 11.9 and 43 years. The mean hospital stay and median hospital stay were 5.8 ± 3.1 and 6 days. The mean discharge creatinine and median discharge creatinine were 106 ± 28.5 and 104 mmol/l.

Table 5 showed the absolute number of patients on renal replacement therapy in Brunei since the inception of the transplant program in 2013. The percentage increments for transplant (+36%) and peritoneal dialysis (PD) (+98%) have outpaced those for HD (+14%) significantly in the last seven years.

Table 6 demonstrated the type of kidney transplants that Brunei Nationals had in the past 35 years with a significant downward trend for foreign kidney transplant commercialization, particularly since the inception of the program in 2013.

## 4. Discussion

The journey started with a vision of a universally accessible local transplant program that caters to the needs of all residents of the country. Previously all transplant patients (citizens of the country only) had the option of being sent overseas for transplantations if there was a compatible donor. The patient would typically stay abroad for 3–6 months. The absence of a local program meant that many eligible patients have forfeited transplant opportunities because they had not been able to take time away from family and work. Additionally, permanent and temporary residents of the country (which comprised 30% of the population⁵) were not entitled to have government-funded treatment. The vision of an independent program was only able to come to fruition when a local team took the initiative to instigate a pioneering project to start a local transplant program ten years ago.

From the outset, the project was divided into four phases. Each phase was meticulously planned, and specific objectives and targets were established and evaluated at regular intervals. The phases are described in Table 6 and subsequent sections.

### 4.1. Phase 1: Gathering Evidence for Feasibility, Sustainability, and Cost-Effectiveness (2009–2012)

The team wanted to answer several key questions that were specific to the needs of the country. A study in 2010 involving 300 participants (approximately 0.1% of the country's eligible population) demonstrated that 78.7% were willing to donate their kidneys if the need arose. 59.7% wanted to have a local program in the country [18]. Another project on the quality of life involving 129 patients confirmed that transplant patients had a better quality of life than HD and PD patients using the World Health Organization Quality of Life (WHOQOL-BREF) questionnaire [19]. A 2012 study, done to assess the graft and patient survival rates of all transplant patients over twenty years (1993 to 2012) [8], showed that the 5- and 10-year patient survival of 93.3% and 90.1% were equivalent to rates achieved in most developed countries. This study demonstrated that the local team has the capacity to look after long-term patients. Lastly, a cost-effectiveness study in 2012 showed that having taken into account the costs of maintenance immunosuppressive treatment, hospital stays, and operation fees, the transplant was more cost-effective than dialysis in the first year. The discrepancy in costings between the two modalities widened considerably after the first year of transplant (which included costs of sending patients overseas).

### 4.2. Phase 2: Assembling Core Local Team, Compiling Transplant Dossier, and Forging Foreign Affiliations (2012–2013)

Phase 2 was concerned with assembling and empowering a core local team to spearhead the project on various fronts, especially to address the multitude of nonclinical issues including ethics, law, religion, and policy.

To assemble a local team, we enlisted three experienced local surgeons—a general surgeon, urologist, and vascular surgeon and deliberately fragmented the transplant surgery into different parts to cater to their existing expertise. The intentions were to have the urologist retrieving the donor's kidney and performing the ureteric anastomosis, the vascular surgeon operating the vascular anastomosis, and the general surgeon anchoring the entire procedure and preparing the transplant vascular bed. As the three surgeons were very experienced, it was felt that they could acquire the required skills with less exposure and training than a freshly trained surgeon performing an entire unfragmented procedure. Numerous studies have shown that experienced general surgeons and urologists can have safe transplant outcomes with limited training exposure [20–22]. Additional team members, including a nephrologist and transplant coordinator, were exposed to structured training in the United Kingdom specifically for training on the work-up process, inpatient management, postoperative follow-up, preparing policy papers, and running public workshops to improve public awareness.

The team worked with the lawyers from the Attorney General Office in drafting the National Transplant Act, which primarily focused on the prohibition and penalization of organ trafficking and commercialization. Enactment of the Transplant Act is anticipated to take a few years as it required sanctioning from parliamentary offices. Interim measures through strict policy regulations with emphasis on compliance to the Istanbul Declaration of Organ Trafficking and Transplant Tourism (DOI) [23] were necessary to safeguard the integrity of transplant activities in the country. As transplants have never been performed in the country, there were uncertainties about whether it was permissible under Islamic laws to donate. Experiences from other Muslim countries especially Malaysia were drawn and used to support our stance on transplantation, especially in using the mosques as the gateways into the Muslim community for impartment of knowledge about organ donation [24]. Through continuous dialogue, engagement, and consultation, we were able to convince the local religious authorities to issue a religious decree (fatwa) to legalize transplantation in the country. We also had to assist the MOH in the setting up of the National Transplant Ethics Committee (NTEC) and the National Transplant Committee (NATCOM). NTEC is concerned with evaluating and providing approval for transplants in accordance with the ethical principles laid out by the DOI and NATCOM supports and oversees the operational aspects and provides a recommendation to the MOH for any transplant-related activities.

Numerous models for international transplant partnerships were studied and scrutinized. The International Society of Nephrology and The Transplantation Society sisters transplant program created new transplant centers and developed existing kidney transplant programs in developing countries like Guatemala, Palestine, Myanmar, Tanzania, and Armenia [25]. These centers received funding from ISN and TTS to develop and support training links. Transplant Links Community (TLC), a UK charitable organization, has also made commendable efforts in establishing transplant activities in Barbados, Zambia, Jamaica, Bangladesh, Nigeria, Kenya, Ghana, and Trinidad [26]. Mongolia started its program after sending a core team of clinicians to Thailand, China, United Kingdom, and Finland to receive intensive training in leading transplant centers [16]. Similarly, Myanmar started its transplant program with the assistance of a joint international team, having sent doctors and nursing staff for proper training abroad [13]. Tapping in on the experience and success of these models, we explored transplant establishments in many countries but eventually decided to forge a working partnership with a Malaysian private hospital that utilized the service of a visiting Australian transplant surgeon.

### 4.3. Phase 3: Experiential Exposure and Strengthening Local Foundation (2012)

This phase was concerned with getting the team experienced and the local infrastructure and manpower ready for the incipient program. We started sending patients with our medical team to our allied foreign center in 2002 to obtain experiential exposure in surgical, medical, and nursing training. The team stayed with the patient for the duration of the hospital stay abroad until the patient was deemed fit for discharge to Brunei.

With a focus on strengthening local facilities, particular attention was given to environmental infection aversion strategies and ventilation engineering controls to prevent transmission of infections. Through guidance from our infection control team, we were able to introduce purpose-built ward facilities and procure consumables to enable the proper use of disinfectants and cleaners and maintenance of medical equipment. We also established new services to the laboratory in preparation for the program like calcineurin inhibitor assays, nuclear medicine scan, bone density scan, and new medications into our formulary. For services that we cannot implement, we sent tests to overseas laboratories in Singapore and Malaysia for crossmatching (CDC, Luminex, and flow cytometry), viral assay, and everolimus assay.

We took into account the varied contributions of members of the interdisciplinary team and their potential impact on transplant outcomes and enhancing living kidney donations. Special efforts were made to conciliate their roles and instill an ethos of shared program ownership. Allied healthcare professionals were given special emphasis in their training through MOH-endorsed workshops, designed to help them integrate and identify with the vision of the team and to facilitate their involvement during the workup and follow-up phases.

### 4.4. Phase 4: Implementation and Consolidation of Transplant Program (2013 to Current)

The first transplant in Brunei was performed on 21/11/2013. A transplant team comprising a physician, a surgeon, and two nurses were flown in from Malaysia and Australia to guide and assist with the procedures. By the third transplant, we had acquired self-sufficiency in many areas and, only the Australian surgeon continued to come to the country at that point. The initial few years of the program were hampered by inadequate staffing and lukewarm interest from the public. As we progressed with a few successful stories (that we had deliberately played out in the press), the public began to register more interest. By the tenth transplant, the team was deemed to have acquired full proficiency in independently performing the transplants and running the program.

Throughout the course of several transplants, we were able to gradually improve and introduce new services to the program (Table 3), through numerous audits and quality improvement projects. To maintain self-sufficiency and governance, we have an active research unit that had churned out several review articles on transplantation between 2017 and 2019. This was to enable the team to acquaint themselves with transplant literature and keep abreast with the latest transplant developments. Review articles have been published on aspirin usage in kidney transplants [27], hematological cytopenia in kidney transplants [28], use of fluoroquinolone in BK virus nephropathy [29], cigarette smoking in kidney transplants [30], and dual kidney transplantations [31]. Another recent local-driven study in 2017 showed that lack of donors (71%), lack of awareness of the program (21.2%), and unwillingness to take risks (26.5%) were the main barriers to the progress of the program [32].

Our recipient stayed in the hospital with a mean stay of 15 days which is higher than 6–12 reported in the literature [33]. This may reflect extra vigilance in a newly set-up transplant program. One-year patient survival and graft survival were 100%. Three of our patients reached 5 years, and graft and patient survival was 100%. Our results are comparable to those reported in other studies [34]. We did not observe major complications in our donors. Only three have a minor complication. We noticed 20% rejections (2/10) which are comparable to those reported of 15–20% from various centers [35]. These findings reflect the cautious establishment of the new kidney transplant program under the supervision of an experienced and trained transplant surgeon along with the local team and maintaining safety at the same time. We expect that our number will go up in the near future. To garner public interest, we have initiated quarterly seminars with potential future recipients and donors to discuss the risks and benefits of transplantations with a focus on autonomy, benevolence, and nonmaleficence. We put an emphasis on previous transplant recipients and donors conducting the lectures and seminars, specifically addressing local issues like religion, family apprehensions, and the danger of commercialization. We acknowledge few limitations of our study. Our numbers are low. Due to retrospective data collection, we missed important data like cold ischemia time and donor operation time.

## 5. Conclusion

The challenges that we faced were unique and covered many nonclinical areas that were not normally encountered by established centers. We had to overcome technical, religious, cultural, political, and legislative barriers to inaugurate our national program. As stated from the outset, the core principles of the program were hinged around the ideals of equity, quality, sustainability, and morality; henceforth, the pace of the fledging program can only be determined by the slow disentanglement of existing limitations to enable the successful fulfillment of these virtues. It has been a truly enriching and gratifying journey, and we hope that our experience can inspire and benefit other new centers, especially in small and developing countries.

## Figures and Tables

**Table 1:** Recent transplant programs in Asian countries.

Country	Year started	Population (millions)	Size (km^2^)	GDP per capita (USD)	Living-related transplant (LRT)	Cadaveric	Progress
Myanmar	1997	54.05	653,290	1,286	Yes	Yes	22 transplants between 1997 and 2003 > 50 transplants between 1997 and 2012
Armenia	2002	2.95	28,470	3,918	Yes	Unknown	129 LRT performed between 2002 and 2016
Mongolia	2006	3.22	1,553,560	3.672	Yes	Yes	213 transplants including 13 cadaveric transplants from 2006 to 2016
Nepal	2008	28.61	143,350	900	Yes	Yes	517 LRT performed between 2008 and 2018
Tajikistan	2009	9.32	139,960	805	Yes	Unknown	104 transplants in 2018
Uzbekistan	2010	32.98	425,400	1,554	Yes	Unknown	Unknown
Kyrgyzstan	2012	6.41	191,800	1,222	Yes	Unknown	Unknown
Brunei	2013	0.44	5,270	28,572	Yes	No	10 transplants performed in 6 years, with 5 in the last two years

**Table 2:** Demographic, operative, and hospital stay details for recipients.

	Gender	Relationship	Age at transplant	Age at ESRD	Year of operation	Diagnosis	Blood group	Presence of DSA	HLA mismatch	Panel reactive antibody (%)	Warm ischemia time (minutes)	Operation time (minutes)	Surgical complications	Hospital stay (days)
1	Female	Daughter	21	19	2013	Henoch schonlein nephritis	O	No	1	<3	150	236	Bleeding intraoperatively from venous anastomosis. Received 4 units of blood	12
2	Male	Son	29	28	2014	Unknown	AB	No	2	47	90	213	—	14
3	Male	Husband	45	42	2015	Unknown	B	No	5	<3	90	297	—	16
4	Female	Sister	28	25	2016	Unknown	B	DR52 (MFI 668)	0	<3	75	280	—	15
5	Female	Daughter	26	25	2017	Unknown	AB	No	2	<3	153	290	—	13
6	Female	Wife	29	28	2018	Unknown	A	DQ6 (MFI 4514)	5	Class I 11, class II <3	105	225	—	15
7	Male	Brother	47	47	2018	Mesangiocapillary glomerulonephritis	A	No	0	Class I 4, class II 10	120	226	Rectus sheath haematoma which settled with conservative management	16
8	Female	Sister	23	23	2019	Unknown	B	DRB3 (MFI 768)	3	<3	141	315	—	13
9	Male	Brother	36	32	2019	Unknown	O	No	0	Class I <3, class II 38	73	254	Surgical reexploration for infected wound haematoma	23
10	Male	Brother	21	20	2019	Focal segmental glomerulosclerosis	A	No	2	<3	132	341	—	13

**Table 3:** Follow-up details of recipients.

Recipient	Duration of follow-up (months)	Discharge creatinine (mmol/l)	1 years creatinine (mmol/l)	2 years creatinine (mmol/l)	5 years creatinine (mmol/l)	CMV	BK	Biopsy	Biopsy proven rejection	Mycophenolate mofetil	Mycophenolate sodium	Tacrolimus	Everolimus	Services introduction
1	72	105	104	101	137	Yes	—	Yes	—	Yes	—	Yes	—	Tacrolimus assay
2	61	139	133	138	152	—	—	—	—	Yes	—	Yes	Yes	—
3	51	159	152	147	—	—	—	Yes	—	Yes	—	Yes	Yes	—
4	43	92	91	64	—	—	—	—	—	—	—	—	Yes	—
5	28	120	134	152	—	—	—	Yes	—	—	Yes	Yes	—	Renal HDU
6	21	86	83	—	—	—	—	Yes	Yes	Yes	—	Yes	—	Induction with ATG
7	15	82	88	—	—	—	Yes	—	—	Yes	—	Yes	—	Everolimus assay
8	6	75	—	—	—	—	—	Yes	—	Yes	—	Yes	—	DTPA
9	6	87	—	—	—	Yes	Yes	—	—	—	Yes	Yes	Yes	—
10	0	120	—	—	—	—	—	Yes	Yes	Yes	—	Yes	—	—

**Table 4:** Demographic, operative, and hospital stay details for donors.

	Gender	Relationship	Age at surgery/donation	Year of operation	Blood group	Donor kidney description	Hospital stay (days)	Discharge creatinine (mmol/l)	1 year creatinine (mmol/l)	Last follow-up
1	Male	Father	45	2013	O	Left kidney with two renal veins	4	104	110	(i) Creatinine 107 mmol/l on the last follow-up in April 2020
										(ii) Has diet controlled dyslipidemia
										(iii) No evidence of hypertension, proteinuria, or diabetes

2	Male	Father	54	2014	B	Left kidney	5	140	156	(i) Creatinine 139 mmol/l in October 2020
										(ii) No evidence of hypertension, proteinuria, or diabetes

3	Female	Wife	45	2015	O	Left kidney	5	77	96	(i) Creatinine 96.9 mmol/l during last follow-up in August 2020
										(ii) No evidence of hypertension, proteinuria, or diabetes

4	Female	Sister	26	2016	B	Left kidney	6	66	65	(i) Creatinine 76.9 mmol/l during last follow-up in September 2020
										(ii) No evidence of hypertension, proteinuria, or diabetes

5	Male	Father	53	2017	A	Left kidney with two renal arteries	6	140	141	(i) Creatinine 146 mmol/l during the last follow-up in September 2020
										(ii) Has diet controlled dyslipidemia
										(iii) No evidence of hypertension, proteinuria, or diabetes

6	Male	Husband	31	2018	O	Left kidney	8	131	146	(i) Creatinine 146 mmol/l during last follow-up in September 2020
										(ii) On atorvastatin for dyslipidemia
										(iii) No evidence of hypertension, proteinuria, or diabetes

7	Male	Brother	43	2018	A	Left kidney	7	127	140	(i) Creatinine 130 mmol/l during last follow-up in April 2020
										(ii) Has two episodes of gout and diet-controlled dyslipidemia
										(iii) No evidence of hypertension, proteinuria or diabetes

8	Female	Sister	22	2019	B	Left kidney	6	75	77	(i) Creatinine 78 mmol/l during last follow-up in December 2020
										(ii) No evidence of hypertension, proteinuria, or diabetes

9	Male	Brother	43	2019	O	Right kidney	5	119	—	(i) Creatinine 135 mmol/l during the last follow-up in September 2020
										(ii) No evidence of hypertension, proteinuria, or diabetes

10	Female	Sister	24	2019	A	Left kidney with two arteries	6	87	—	(i) Creatinine 86.8 mmol/l during the last follow-up in September 2020
										(ii) No evidence of hypertension, proteinuria, or diabetes

**Table 5:** Modalities of RRT in Brunei and percentage increases since 2013.

	Transplant	HD	PD	All RRT
2013	36	570	46	652
2014	39	606	53	698
2015	45	586	67	698
2016	47	629	78	754
2017	47	656	75	778
2018	46	655	82	783
2019	49 (+36%)^*∗*^	651 (+14%)^*∗*^	83 (+98%)^*∗*^	783 (+20%)^*∗*^

^*∗*^- denotes the % increase since 2013.

**Table 6:** Phases of the kidney transplant program in Brunei.

Phases	Brief description	Subphases	Description
Phase 1	Gathering evidence	1 (a) public opinion study	78.7% of the public were willing to donate and 59.7% trusted a local program
		1 (b) quality of life study	Better physical health, psychological, social relationship, and environment scores in transplant patients
		1 (c) graft survival study	5 years and 10 years graft survival of 93.1% and 90.1% were better than most countries
		1 (d) cost-effectiveness study	The transplant was cheaper than dialysis. Transplant in Brunei is cheaper than sending patients abroad

Phase 2	Assembly of the core team, compiling transplant dossier and forging foreign affiliations	2 (a) assembling core team	Core team comprising local nephrologists, surgeons, transplant coordinators, and nurses
		2 (b) drafting of National Transplant Act	Focus on prohibition and penalization of organ trafficking and commercialization
		2 (c) issuance of religious decree (fatwa)	The decree legitimizes the act of donation and receiving transplant under Muslim laws
		2 (d) constitutions of national transplant-related committees	The National Transplant Committee (NATCOM) and Transplant Ethics Committee (NTEC) deal with policy, ethical, and governance issues that arise with transplant
		2 (e) formulation of national transplant policy	The policy gives guidance on how to uphold the standards of transplant in the country
		2 (f) forging of foreign affiliations	The signing of the memorandum of understanding with Prince Court Medical Center, Malaysia

Phase 3	Experiential exposure and strengthening the local foundation	3 (a) enhancing experiential experience	Hands-on training for key staff in Prince Court Medical Center
		3 (b) strengthening hospital facilities	Improvement of the ward, operating theatre, and laboratory facilities
		3 (c) bespoke training for allied healthcare professionals	Special training through workshops and seminars conducted for all associated healthcare professionals by local and foreign experts
		3 (d) introduction of transplant subsidiary services	Numerous new laboratory, radiological, and pharmacological introductions to the services. Partnership with foreign institutions for superspecialized services like crossmatch and viral assays

Phase 4	Implementation and consolidation	4 (a) implementing program	The main goal is to implement a program that is equitable, safe, sustainable, and morally sound
		4 (b) dynamic auditing and reviewing of services	Constant reviewing of the services led to the introduction of new services to modernize services and eradicate errors
		4 (c) maintaining self-sufficiency and governance through research	Many review articles and research have been published on transplant topics by the core team during this phase
		4 (d) public awareness campaigns	Regular seminars and workshops to garner and maintain public interest

## Data Availability

The retrospective data used to support the findings of this study are included within the article.
